# The mechanisms by which antidepressants may reduce coronary heart disease risk

**DOI:** 10.1186/s12872-015-0074-5

**Published:** 2015-08-01

**Authors:** Marc J. Mathews, Edward H. Mathews, Leon Liebenberg

**Affiliations:** grid.25881.360000000097692525CRCED Pretoria, North-West University, P.O. Box 11207, Silver Lakes, 0054 South Africa

## Abstract

**Background:**

Depression is known to increase the risk for coronary heart disease (CHD) likely through various pathogenetic actions. Understanding the links between depression and CHD and the effects of mediating these links may prove beneficial in CHD prevention.

**Methods:**

An integrated model of CHD was used to elucidate pathogenetic pathways of importance between depression and CHD. Using biomarker relative risk data the pathogenetic effects are representable as measurable effects based on changes in biomarkers.

**Results:**

A ‘connection graph’ presents interactions by illustrating the relationship between depression and the biomarkers of CHD. The use of selective serotonin reuptake inhibitors (SSRIs) is postulated to have potential to decrease CHD risk. Comparing the ‘connection graph’ of SSRI’s to that of depression elucidates the possible actions through which risk reduction may occur.

**Conclusions:**

The CHD effects of depression appear to be driven by increased inflammation and altered metabolism. These effects might be mediated with the use of SSRI’s.

## Background

Depression is one of several preventable causes of disability worldwide, with coronary heart disease (CHD) being the largest cause of disability [1]. In addition, CHD is also the largest cause of death globally [2].

There is an established link between these two disorders, where depression has been noted as a risk factor for CHD [3] and patients with established CHD have been found to have increased incidence of depression compared to controls [4]. Depressed CHD patients are significantly linked to increased mortality [5] and poor prognosis for further CHD events [6]. Depressed patients using antidepressants appear to be at a reduced risk for CHD. However, the mechanisms behind this reduced risk are not clear [7].

To gain more insight into associations between depression, antidepressants, and CHD an integrated model of CHD pathogenesis, health factors, biomarkers and pharmacotherapeutics would be beneficial [8]. We can then consider the effect of treatment of depression with antidepressants on the pathogenesis of CHD. This will help with insight as to how antidepressants might decrease CHD risk in the depressed.

## Methods

### Health factor integration with CHD

Our integrated model was developed and described in a previous article [9]. Briefly, a systematic review of the literature from after 1998 and including highly cited papers was conducted for CHD pathogenesis, health factors, biomarkers and pharmacotherapeutics. This research was combined to develop the integrated model of CHD [9].

The health factors in the integrated model were considered as lifestyle effects or comorbid health disorders which have been associated with statistically significant increases or decreases in CHD risk. The pharmaceuticals in the integrated model were those whose use has been associated with statistically significant decreases in CHD risk in primary or secondary prevention.

The biomarkers considered for the integrated model were mainly those whose measurement has been associated with statistically significant increases or decreases in CHD risk. However, some biomarker data was included where results have not been statistically significant as an emphasis of their lack of prediction ability.

The above components were combined to develop the integrated model [9] which will be used in this article to describe the interconnections of depression on the pathogenesis of CHD. We attempt to quantify the CHD effect of depression and antidepressants by the effect thereof on an array of biomarkers which represent increasing or decreasing CHD risk. The study dealt mainly with the primary prevention aspects as most of the data gathered for the effects of SSRI use on the biomarkers was from studies in patients without CHD.

### Statistical analysis

It must be noted that some of the RR values in this article are presented in a manner which differs from convention [9]. The need for this comes as a result of the visual scaling of the traditional RR. Traditionally, if one plots an RR = 3 and RR = 0.33, respectively, the one does not ‘look’ three times worse and the other three times better than the normal RR = 1. The reason is that the scales for the positive and negative effects are not numerically similar. A graph of ‘good’ and ‘bad’ RR can therefore be deceptive for the untrained person, e.g., a patient.

This article rather uses the method that the conventional RR = 3 is three times worse than the normal RR = 1. While the conventional RR = 0.33 means that the patient’s position is three times better than the normal RR = 1. Thus, in summary: a conventional RR = 3 is presented as per normal, as a 3-fold increase in risk and a conventional RR = 0.33 is presented as a 3-fold decrease in risk (1/0.33 = 3).

## Results

### Integrated model

**Fig. 1:**
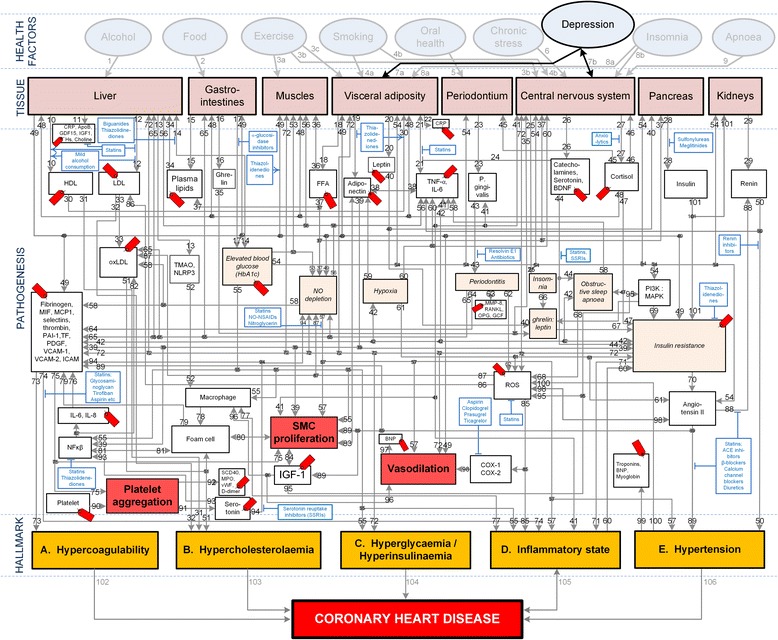
Conceptual model of general health factors, salient CHD pathogenetic pathways and CHD hallmarks. Note. From “How do high glycemic load diets influence coronary heart disease?” by Mathews M, Liebenberg L, Mathews EH *Nutr Metab* 2015;12:6 [9]. The affective pathway of pharmacotherapeutics, boxes, is shown in Fig. 1, and salient serological biomarkers are indicated by tags (). The blunted arrows denote antagonize or inhibit and pointed arrows denote up-regulate or facilitate. ACE denotes angiotensin-converting-enzyme; BDNF, brain-derived neurotrophic factor; β-blocker, beta-adrenergic antagonists; BNP, B-type natriuretic peptide; COX, cyclooxygenase; CRP, C-reactive protein; D-dimer, fibrin degradation product D; FFA, free fatty acids; GCF, gingival crevicular fluid; HDL, high-density lipoprotein; HbA1c, glycated hemoglobin A1c; Hs, homocysteine; ICAM, intracellular adhesion molecule; IGF-1, insulin-like growth factor-1; IL, interleukin; LDL, low-density lipoprotein; MAPK, mitogen-activated protein (MAP) kinase; MCP, monocyte chemoattractant protein; MIF, macrophage migration inhibitory factor; MMP, matrix metalloproteinase; MPO, myeloperoxidase; NFκβ, nuclear factor-κβ; NLRP3, Inflammasome responsible for activation of inflammatory processes as well as epithelial cell regeneration and microflora; NO, nitric oxide; NO-NSAIDs, combinational NO-non-steroidal anti-inflammatory drug; OPG, osteoprotegerin; oxLDL, oxidized LDL; P. gingivalis, Porphyromonas gingivalis; PAI, plasminogen activator inhibitor; PDGF, platelet-derived growth factor; PI3K, phosphatidylinositol 3-kinase; RANKL, receptor activator of nuclear factor kappa-beta ligand; ROS, reactive oxygen species; SCD-40, recombinant human sCD40 ligand; SMC, smooth muscle cell; SSRI, serotonin reuptake inhibitors; TF, tissue factor; TMAO, an oxidation product of trimethylamine (TMA); TNF-α , tumor necrosis factor-α; vWF, von Willebrand factor; VCAM, vascular cell adhesion molecule

The integrated model in Fig. 1 schematically illustrates the complexity of CHD and shows all theoretical pathogenetic pathways between the health factors and CHD. The health factors that are described by the integrated model include both modifiable lifestyle effects and underlying comorbid disorders such as depression. A more detailed discussion of Fig. 1, relevant to depression, is given in next section.

The pathways (pathogenesis of CHD) within the integrated model can be tracked from where a chosen health factor influences the relevant tissue, to the end state of CHD. This will be conducted for depression in the following section of this study. The pathways presented in Fig. 1 are a visual representation of previously published knowledge. Salient serological biomarkers (shown in Fig. 1 as ) and pharmacotherapeutics (shown in Fig. 1 as ) that act on the pathways are further indicated in Fig. 1.

### Pathogenetic effects of depression

In order to appraise the CHD effects of depression, the relevant pathogenetic pathways need to be considered. While Fig. 1 also indicates other health factors, only the pathways activated by depression, presented in Fig. 1, are summarized in Table 1. It is important to note that not all of the pathways will be relevant to every patient and that all the pathways may not be active simultaneously, or occur in the same patient.

**Table 1:** Putative effects of depression and salient CHD pathogenetic pathways

Pathways, and pathway numbers corresponding to those in Fig. 1	Refs.
a. 7-26-↑ catecholamines/↓ serotonin/↓ BDNF-44-↑ platelet factors-73-↑ hypercoagulability	a. [95–97]
b. 7-26-↑ catecholamines/↓ serotonin/↓ BDNF-44-↑ NO depletion-57-↑ SMC proliferation	b. [95]
c. 7-26-↑ catecholamines/↓ serotonin/↓ BDNF-44-↑ NO depletion-57-↑ vasodilation	c. [95]
d. 7-26-↑ catecholamines/↓ serotonin/↓ BDNF-44-↑ insulin resistance-70-↑ angiotensin II-89-↑ hypertension-100-↑ ROS-85-↑inflammatory state	d. [95, 98–103]
e. 7-26-↑ catecholamines/↓ serotonin/↓ BDNF-44-↑ insulin resistance-70-↑ angiotensin II-88-50-↑ TNFα-41-↑ inflammatory state	e. [44, 104–108]
f. 7-26-↑ catecholamines/↓ serotonin/↓ BDNF-44-↑ insulin resistance-70-↑ angiotensin II-89- ↑ SMC proliferation	f. [95, 99, 101–103, 109]
g. 7-26-↑ catecholamines/↓ serotonin/↓ BDNF-44-↑ insulin resistance-70-↑ angiotensin II-89-↓ IGF1-↑ SMC proliferation	g. [101–103, 109, 110]
h. 7-26-↑ catecholamines/↓ serotonin/↓ BDNF-44-↑ insulin resistance-72-↑ platelet factors-73-↑ hypercoagulability	h. [17, 29, 99, 110–117]
i. 7-26-↑ catecholamines/↓ serotonin/↓ BDNF-44-↑ insulin resistance-72-14-55-↑ hyperglycaemia	i. [110, 118–120]
j. 7-26-↑ catecholamines/↓ serotonin/↓ BDNF-44-12-↑ LDL-33-↑ oxLDL-51-↑ hypercholesterolaemia	j. [29, 95, 121, 122]
k. 7-26-↑ catecholamines/↓ serotonin/↓ BDNF-44-↑ insulin resistance-70-↑ angiotensin II-89-↑ hypertension-100-↑ ROS-85-↑ inflammatory state	k. [95, 106–108]
l. 7-27-↑ cortisol-48-10-↓ HDL-31-↑ hypercholesterolaemia	l. [14, 17, 29, 99]
m. 7-27-↑ cortisol-48-12-↑ LDL-33-↑ oxLDL-51-↑ hypercholesterolaemia	m. [14, 17, 29, 98, 99]
n. 7-27-↑ cortisol-48-14-↑ blood glucose-55-↑ hyperglycaemia	n. [14, 17, 29, 99]
o. 7-27-↑ cortisol-48-14-↑ blood glucose-54-69-↑ insulin resistance-70-↑ angiotensin II-89-↑ hypertension-100-↑ ROS −85-↑ inflammatory state	o. [98–100]
p. 7-27-↑ cortisol-48-14-↑ blood glucose-54-69-↑ insulin resistance-70-↑ angiotensin II-88-50-↑TNFα-41-↑ inflammatory state	p. [123]
q. 7-27-↑ cortisol-48-14-↑ blood glucose-54-69-↑ insulin resistance-70-↑ angiotensin II-89-↑ SMC proliferation	q. [99]
r. 7-27-↑ cortisol-48-14-↑ blood glucose-54-69-↑ insulin resistance-70-↑ angiotensin II-89-↓ IGF1-↑ SMC proliferation	r. [101–103, 109]
s. 7-27-↑ cortisol-48-14-↑ blood glucose-54-69-↑ insulin resistance-72-↑ platelet factors-73-↑ hypercoagulability	s. [17, 29, 99, 111–117]
t. 7-27-↑ cortisol-48-14-↑ blood glucose-54-69-↑ insulin resistance-72-↑ vasodilation	t. [123]
u. 7-27-↑ cortisol-48-14-↑ blood glucose-54-19-↓ adiponectin-38-↑ TNFα-41-↑ P.gingivalis-43-↑ periodontitis-64-↑ platelet factors-73-↑ hypercoagulability	u. [17, 29, 99, 111–117, 124]
v. 7-27-↑ cortisol-48-14-↑ blood glucose-54-19-↓ adiponectin-39-↑ insulin resistance- 72-↓ vasodilation	v. [123]
w. 7-27-↑ cortisol-48-14-↑ blood glucose-54-19-↓ adiponectin-39-↑ SMC proliferation	w. [125]
x. 7-27-↑ cortisol-48-14-↑ blood glucose-54-↑ PI3K:MAPK-69-↑ insulin resistance-72-14-55-↑ hyperinsulinaemia	x. [17, 20, 29, 99]
y. 7-27-↑ cortisol-48-14-↑ blood glucose-53-↑ NO depletion-57-↑ SMC proliferation	y. [17, 29, 99, 126]
z. 7-27-↑ cortisol-48-14-↑ blood glucose-53-↑ NO depletion-57-↓ vasodilation	z. [17, 29, 99, 127]
aa. 7-27-↑ cortisol-48-14-↑ blood glucose-54-↑ angiotensin II-89-↑ hypertension-100-↑ ROS-85-↑ inflammatory state	aa. [17, 29, 98, 99]

↑ denotes up regulation/increase, ↓ denotes down regulation/decrease, x-y-z indicates pathway connecting x to y to z

FFA free fatty acids, IGF 1 insulin-like growth factor-1, LDL low-density lipoprotein, MAPK mitogen-activated protein (MAP) kinase, NO nitric oxide, oxLDL oxidized LDL, P. gingivalis Porphyromonas gingivalis, PI3K phosphatidylinositol 3-kinase, PI3K:MAPK ratio of PI3K to MAPK, ROS reactive oxygen species, SMC smooth muscle cell, TNFα tumor necrosis factor-α

Some of the pathological effects of depression on CHD are thought to be mediated by the over stimulation of the hypothalamic-pituitary-adrenocortical (HPA) axis [10]. Increased levels of corticotropin-releasing factor (CRF) and its stimulation of the production and release of adrenocorticotropic hormone (ACTH), mediates the activation of the HPA axis [11]. This can lead to increased plasma cortisol levels [12]. The overstimulation of the HPA axis may augment sympathoadrenal (SA) hyperactivity via central regulatory pathways, resulting in increased plasma catecholamines [13], such as norepinephrine, epinephrine and dopamine [14].

Chronic dysregulation of the HPA axis, such as in depression, can lead to chronically increased serum levels of cortisol [12], which can have negative effects on insulin and blood glucose levels [15]. The effect of cortisol on blood glucose is shown in the integrated model (Fig. 1) through *pathway 7-27-48-14-blood glucose-55-hyperglycaemia*, with the possibility that over stimulation of the pathway could lead to the CHD hallmark of hyperglycaemia.

Further, abnormalities in blood glucose control and insulin sensitivity are seen in patients with major depressive disorder, even in individuals who are non-obese and not diabetic [16]. Some of these effects may be explained by the increased secretion of glucocorticoids, which oppose the effects of insulin and increases the turnover between stored energy, in the form of glycogen, triglycerides and protein, and freely available fuel for mitochondrial oxidation, in the form of glucose and free fatty acids [17]. This serves to increase blood glucose levels. Blood glucose levels can also be increased, by glucocorticoids, through an effect on hepatic gluconeogenesis and insulin secretion [15]. (Fig. 1, Pathway: 7-27-48-14-55-hyperglycaemia).

*Pathways: 6-27-47* and *7-26-44* in the integrated model (Fig. 1) show how catecholamines and glucocorticoids inhibit insulin actions and thus contribute to insulin resistance [18, 19]. Additionally, it is possible for insulin resistance to occur due to inhibition of the phosphatidylinositol 3-kinase (PI3K) insulin signaling pathway or the stimulation of the MAPK pathway [20]. (Fig. 1, Pathways: 7-27-48-14-54-69-72-14-55- hyperinsulinaemia).

Elevated glucocorticoids can increase the responsiveness to vasoconstrictors and reduce vasodilator production, noted by a reduction in nitric oxide (NO) production or bioavailability, contributing to glucocorticoid induced hypertension [21]. (Fig. 1, Pathway: 7-27-48-14-53-57-vasodilation).

Another possible mechanism underlying glucocorticoid induced hypertension is shown in the integrated model (Fig. 1) by *pathway: 7-27-48-14-54-89-hypertension.* This details how depression could lead to increased activity of the renin-angiotensin-aldosterone system, high leptin levels and concurrent leptin resistance [22]. Furthermore increased HPA axis activity can also increase oxidative stress along with decreased antioxidant defenses [23], which can lead to increased inflammation [24] as well as lower brain derived neurotrophic factor (BDNF) activity [25]. (Fig. 1, Pathway: 7-27-48-14-54-89-hypertension-100-inflammatory state).

Increased insulin resistance can cause increased serum levels of platelet factors and thus increases the potential for hypercoagulability [26, 27]. Additionally, increased insulin resistance has been found to be associated with increased levels of inflammatory cytokine TNF-a and increased levels of inflammation [28] as shown in the integrated model in *pathway: 7-27-48-14-54-69-70-88-50-41-inflammatory state*.

Elevations in glucocorticoids inhibit lipoprotein lipase activity leading to diminished triglyceride clearance, decreased HDL concentrations, and increase in LDL serum concentrations [29]. Additionally, high levels of glucocorticoids suppress hepatic LDL receptors and delay LDL clearance [30]. This shows how depression can affect cholesterolaemia through *pathways 7-27-48-10-31-hypercholesterolaemia and 7-27-48-12-33-51-hypercholesterolaemia*.

The integrated model shows how depression may affect coagulation and vasodilation through *pathways: 7-26-catecholamines-44-73-hypercoagulability and 7-26-catecholamines-44-57-vasodilation*. Elevated serum levels of catecholamines, such as norepinephrine, may promote hypercoagulability by platelet activation through direct agonist effects, and endothelial injury by increased hemodynamic stress on vascular walls [31].

Decreased levels of BDNF have been observed in depressed patients [32, 33]. Normal or increased levels of BDNF have been found to have positive effects on some of the underlying pathogenesis of CHD including improved glucose metabolism [34]. Thus a reduction of BDNF can thus serve to reduce glucose control, which can have a feedback effect by inhibiting the cerebral output of BDNF [35] as shown in pathway: 7-26-BDNF-44-72-14-55-hyperglycaemia.

However, BDNF may increase oxidative stress through activation of NAD(P)H oxidase [36]. Thus BDNF could have a negative impact on the pathogenesis of CHD and plaque stability. BDNF is thought to positively affect the action and secretion of insulin, ghrelin and leptin [34]. (Fig. 1, Pathway: 7-26-BDNF-44-insulin resistance).

Increased levels of serotonin could serve to up-regulate some of the underlying pathogenesis of CHD. Alterations in serotonergic neuronal function in the central nervous system occur in patients with major depression [37]. Activated platelets secrete serotonin in substantial quantities which can cause vasoconstriction [38]. Additionally, serotonin has a role in platelet aggregation and proliferation of vascular endothelial cells [39, 40]. (Fig. 1, Pathways: serotonin-94-57-SMC proliferation and serotonin-94-57-vasodilation).

It is apparent that depression directly and indirectly affects a plethora of interconnected pathogenetic mechanisms. Each CHD hallmark and pathogenetic trait can amplify the patient’s risk of CHD, thus necessitating an integrated, multi-faceted therapeutic approach.

### Biomarkers of coronary heart disease

While the pathogenesis of depression is not completely understood, the possible pathogenetic effect of depression on CHD could be better understood through the measurement of serological biomarkers [41]. Biomarkers can be used as indicators of an underlying disorder. The measurement of specific biomarkers enables the prediction of the RR for CHD associated with the biomarker [42–44]. This can allow for the quantification of the effects of depression on the pathogenesis of CHD.

**Fig. 2:**
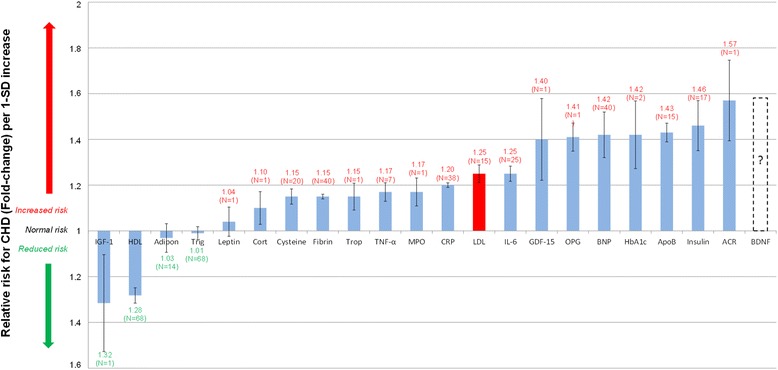
Normalized relative risks (fold-change) of salient current biomarkers or of potential serological biomarkers for CHD. Note. From “How do high glycemic load diets influence coronary heart disease?” by Mathews M, Liebenberg L, Mathews EH Nutr Metab 2015;12:6 [9]. Increased IGF-1 and HDL levels are associated with a moderately decreased CHD risk. (IGF-1 and HDL levels are significantly inversely correlated to relative risk for CHD.) *N* indicates number of trials; I, standard error; ACR, albumin-to-creatinine ratio; Adipo, adiponectin; ApoB, apolipoprotein-B; BDNF, brain-derived neurotrophic factor; BNP, B-type natriuretic peptide; Cort, cortisol; CRP, C-reactive protein; Cysteine, Homocysteine; Fibrin, fibrinogen; GDF-15, growth-differentiation factor-15; HbA1c, glycated hemoglobin A1c; HDL, high-density lipoprotein; IL-6, interleukin-6; IGF-1, insulin-like growth factor-1; LDL, low-density lipoprotein; MPO, myeloperoxidase; RANKL or OPG, osteoprotegerin; TNF-α, tumor necrosis factor-α; Trop, troponins; Trigl, triglycerides

To simplify the integrated model, serological biomarkers (which can be easily measured) are used to link the effect of depression to the corresponding RR of CHD. Figure 2 presents a comparison of the RR associated with an array of serological biomarkers per 1-standard deviation increase in the biomarker [9].

### Effects of depression on coronary heart disease

**Fig. 3:**
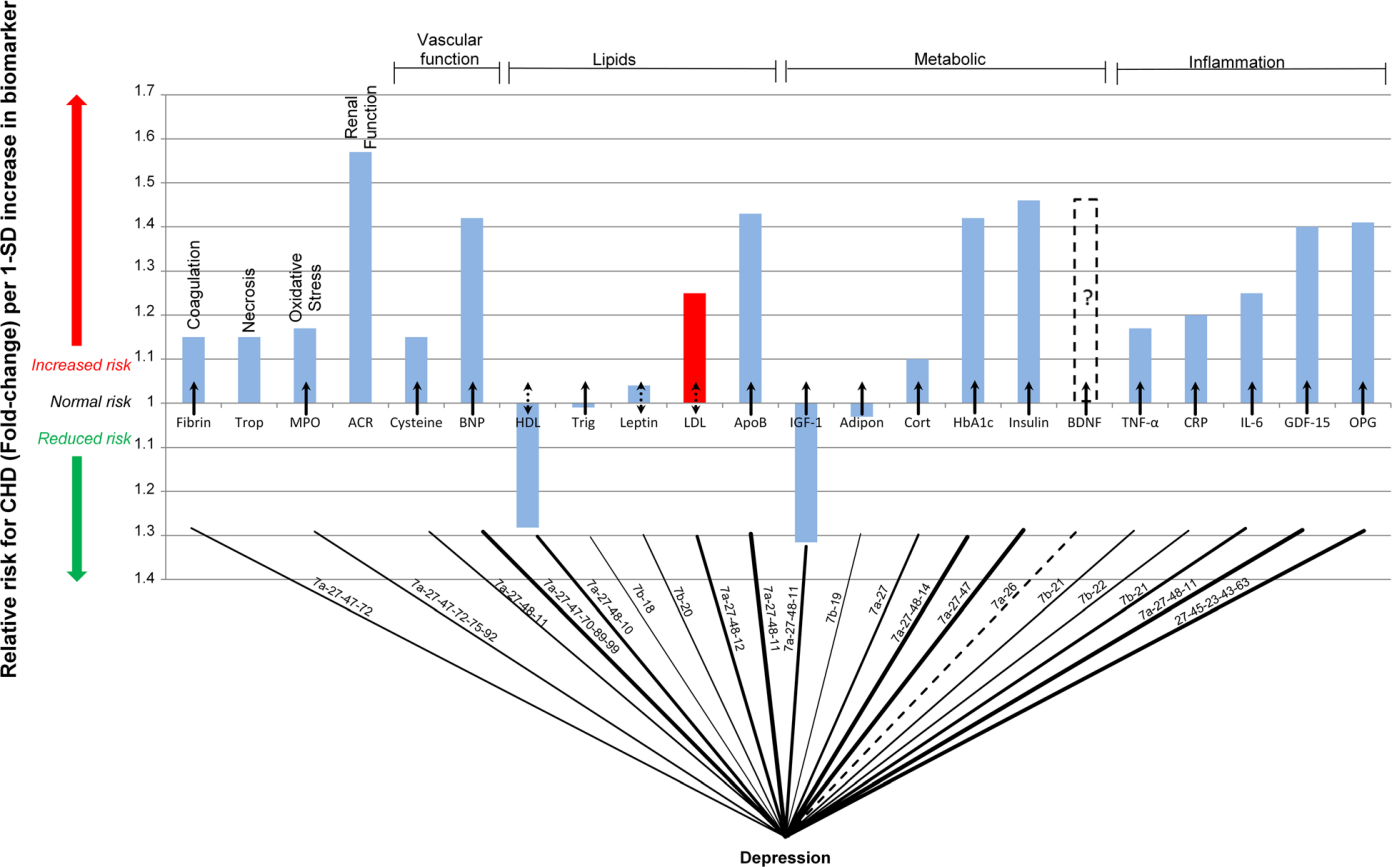
Interconnection of relative risk effects of depression and serological biomarkers for CHD. ACR denotes, albumin-to-creatinine ratio; Adipon, adiponectin; ApoB, Apolipoprotein-B; BDNF, brain-derived neurotrophic factor; BNP, B-type natriuretic peptide; Cort, cortisol; CRP, C-reactive protein; Cysteine, Homocysteine; Fibrin, fibrinogen; GDF-15, growth-differentiation factor-15; HbA1c, glycated hemoglobin A1c; HDL, high-density lipoprotein; IGF-1, insulin-like growth factor-1; IL-6, interleukin-6; LDL, low-density lipoprotein; MPO, myeloperoxidase; RANKL or OPG, osteoprotegerin; TNF-α, tumor necrosis factor-α; Trigl, triglycerides; Trop, troponins

The pathogenesis of depression in CHD and the integrated model in Fig. 1 could be used to account for the impact that depression has on the serological biomarkers of CHD (Fig. 2). The integrated model can be simplified into a ‘connection graph’, which shows all the connections between depression and the serological biomarkers of CHD without neglecting the underlying complexity of CHD. The relevant pathways of Fig. 1 are shown on the connection lines of Fig. 3.

For further clarity the biomarkers previously shown in Fig. 2 were divided into eight classes. Furthermore, the connection lines are scaled according to the RR associated with the biomarker. Thus, the greater the RR for CHD of a biomarker the thicker the connection line will be to that biomarker. For example, the RR for CHD associated with leptin is relatively low, thus the connection line between depression and leptin is thin. The RR for CHD associated with insulin resistance is large thus the connection line between depression and insulin resistance is thick.

While the connection lines give an indication of which biomarkers of CHD are affected by depression they do not indicate the nature of the connection. The effect of the connection are thus shown by arrows in Fig. 3 which indicate whether the effect on the biomarker is to increase (↑) or decrease risk (↓).

The interconnectedness of depression is immediately evident from Fig. 3. Depression is seen to have connections to the vast majority of the CHD biomarkers considered here. It is evident that depression is widely connected to inflammatory and metabolic biomarkers. Additionally, there are connections between all the lipid biomarkers and some of the markers of vascular function, oxidative stress and coagulation.

Increased levels of inflammation have been reported in patients with depression [45, 46]. It has even been suggested that increased inflammatory markers may be a risk factor for the progression of depression [45]. Increased levels of inflammatory markers such as the cytokines CRP, IL-6 and TNF-α have been measured in patients with depression [47, 48], regardless of a causal link between depression and inflammation.

Changes in osteoprotegerin may be possible due to the observation of decreased bone density [49] and an increased risk of osteoporosis in depressed patients [50]. Thus inflammation and depression seem intertwined and could account for some of the increased CHD risk due to depression.

Many of the metabolic aspects of depression could be mediated through the actions of cortisol and BDNF. Increased serum levels of cortisol have been noted in depressed patients [12, 51], and may lead to other metabolic complications such as hyperglycaemia, hyperinsulinaemia and hypercholesterolemia. Thus, BDNF and cortisol may possibly explain the link between depression and glycated hemoglobin (HbA_1c_), insulin resistance, LDL and HDL [15, 19, 29].

BDNF has frequently been found to be reduced in patients with depression with the implication being that reduced levels of BDNF may be a suitable biomarker for depression [52]. Beyond this intriguing possibility for its use as a biomarker for depression it is postulated here that reduced levels of BDNF may also be a suitable prospective biomarker for CHD risk. This is indicated by the dashed bar in Figs. 2 and 3 [53].

Adiponectin levels in patients with depression have been found to be lower than that of healthy controls independent of conventional factors such as coronary heart disease and metabolic disorders [54]. This could imply that lowered adiponectin levels associated with depression could indicate increased risk for CHD.

The connection between depression and the lipid biomarkers is not as clear as between depression and inflammation [55]. Conflicting evidence surrounds the association between depression and cholesterol levels. Some studies have found that HDL, LDL and Apo B levels are increased in patients with depression [56], others have found that depression is associated with decreased HDL and increased LDL levels [57], yet others have found that both LDL and HDL decrease with depression [55]. Regardless of the unknown effect between cholesterol and depression it is evident that there may be some connection between the two.

The effect of depression on other lipid biomarkers such as leptin are also not clearly elucidated as both increased [58] and decreased levels have been noted in patients with depression [59]. Some of the changes in leptin may be mediated to some degree by decrease in BDNF which are observed in depression [60].

The impact of depression on vascular function may be mediated by increased serum levels of homocysteine and B-type natriuretic peptide (BNP) which are evident in patients with major depressive disorder [61, 62]. Increased serum levels of homocysteine and BNP are both associated with an increased risk of CHD [63, 64]. This indicates a possible connection between depression and CHD through an underlying vascular action.

A connection may exist between depression and both oxidative stress and coagulation in the increased levels of serum myeloperoxidase (MPO) and fibrinogen respectively [47, 48]. Thus it is evident that the use of biomarkers may further elucidate the connections between underlying pathogenesis which may be common between both depression and CHD. This may help understanding the relationship between depression and the increased risk for CHD.

### Antidepressants

To attempt to elucidate the effects of antidepressant treatment of depression on the pathogenesis of CHD the integrated model in Fig. 1 was used to formulate a ‘connection graph’ for the use of selective serotonin reuptake inhibitor (SSRI) antidepressants. SSRI’s were chosen as they have been linked to greater likelihood of positive outcome after CHD event [65]. Furthermore, certain other antidepressants, such as tricyclic antidepressants, have been linked to increased incidence of adverse CHD outcomes [66].

**Fig. 4:**
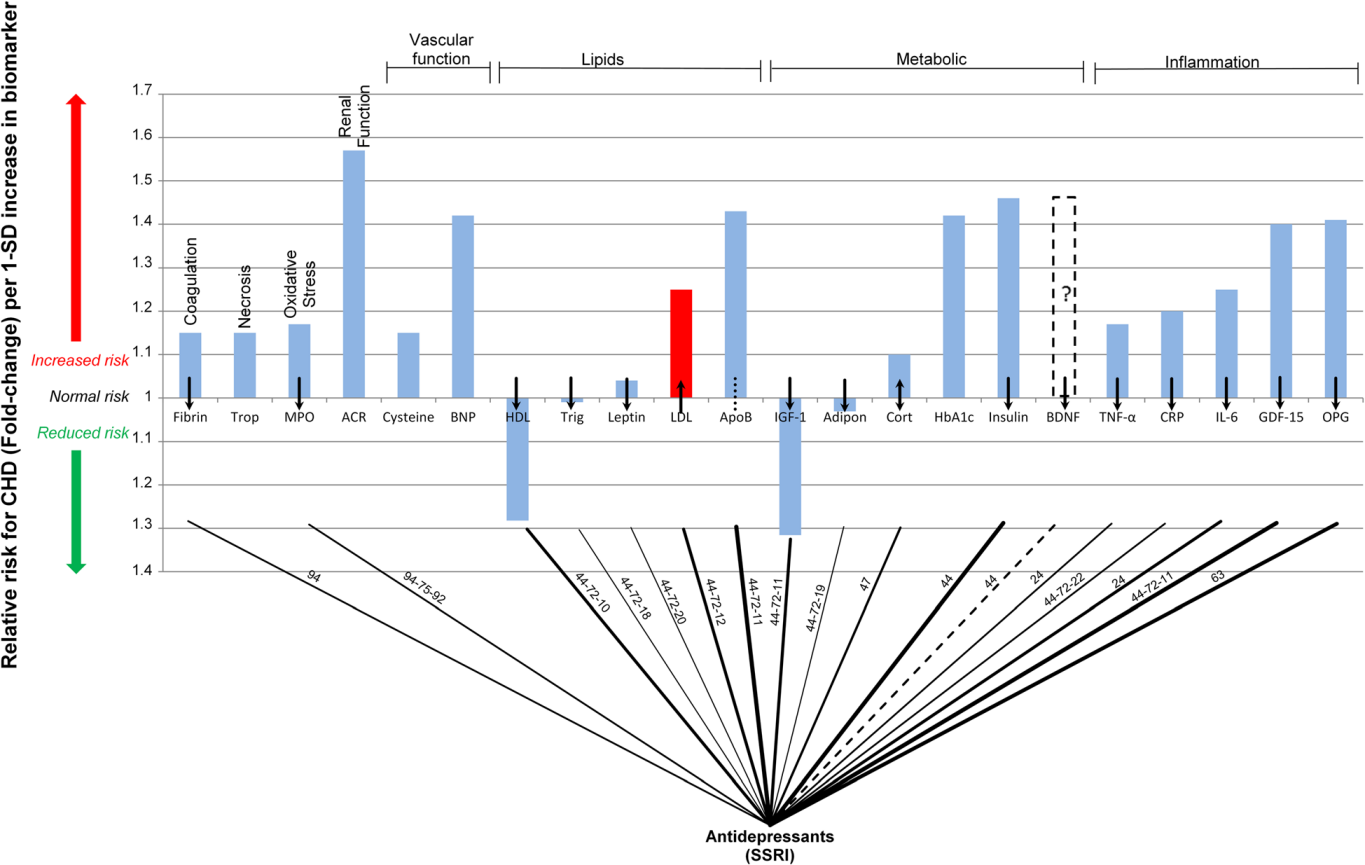
Interconnection of relative risk effects of selective serotonin reuptake inhibitor use and serological biomarkers for CHD. ACR denotes, albumin-to-creatinine ratio; Adipon, adiponectin; ApoB, Apolipoprotein-B; BDNF, brain-derived neurotrophic factor; BNP, B-type natriuretic peptide; Cort, cortisol; CRP, C-reactive protein; Cysteine, Homocysteine; Fibrin, fibrinogen; GDF-15, growth-differentiation factor-15; HbA1c, glycated hemoglobin A1c; HDL, high-density lipoprotein; IGF-1, insulin-like growth factor-1; IL-6, interleukin-6; LDL, low-density lipoprotein; MPO, myeloperoxidase; RANKL or OPG, osteoprotegerin; TNF-α, tumor necrosis factor-α; Trigl, triglycerides; Trop, troponins

The serological biomarkers which are modified by use of SSRI’s are presented in Fig. 4. Figure 4 is a ‘connection graph’ presented in the same manner as was Fig. 3. The ‘connection graph’ for SSRI antidepressants elucidates known changes in serological biomarkers. The paths upon which SSRI’s may act to influence these biomarkers are indicated on the connection lines.

The serum levels of CRP and IL-6 have been observed to be reduced by SSRI use in the depressed [67]. Tumor necrosis factor-α (TNF-α) may play a role in the responsiveness of SSRI use, with increased levels predicting non-responsiveness [68]. The modification of these biomarkers by SSRI’s could serve to decrease the risk for CHD. Osteoprotegerin is decreased by the use of some SSRI’s [69], which may serve to decrease the risk of CHD. SSRI’s affect the entire range of inflammatory biomarkers in a manner that would suggest CHD risk decreases.

The metabolic links between CHD and SSRI’s are most likely mediated by the effect of increased BDNF levels after SSRI treatment [51, 52]. SSRI’s also have an effect on insulin like growth factor 1 (IGF-1) which is low in children using SSRI’s [70] and interruption of SSRI treatment leads to increased serum levels thereof [71]. Increased insulin sensitivity, which has been noted in patients who have remitted depression using SSRI’s [72], could also serve to positively affect serum glucose levels. Increased adiponectin levels have been found to occur due to, inhibition of TNF-α production, after remittance of depression [73].

Cortisol levels have been recorded as both increased [67, 74], and decreased [75] in patients using SSRI’s, thus a possible link exists between SSRI use and serum cortisol levels. However, as a whole the effect of SSRI’s on the metabolic biomarkers would appear to be positive, as shown in Fig. 4. The “connection graph” suggests that the effect of SSRI’s on the metabolic biomarkers is such that it would reduce CHD risk.

The connections between SSRI antidepressants and the lipid biomarkers, shown in Fig. 4, are due to increased serum levels of LDL and HDL cholesterol noted in patients treated with SSRI’s [76, 77]. Current research has shown that serum ghrelin levels can be normalized [78] which could lead to changes in eating habits and thereby affect leptin levels [79]. The net effects of SSRI’s on the lipid profile, in terms a patients risk for CHD, may be somewhat uncertain. This is due to the positive changes in HDL levels, negative changes in LDL levels, no substantial change in leptin levels and an unknown effect on Apo B.

Figure 4 shows the improvements of oxidative stress which may be possible with SSRI [80]. These changes in oxidative stress may be present in patients as changes in MPO serum levels [81]. Furthermore, Fig. 4 shows how serum levels of fibrinogen can be reduced by SSRI use [67]. These changes would present a lower risk for CHD according to biomarker RR prediction.

Unfortunately the fully quantified effect of the different biomarkers, modified by SSRI use, is not shown by the “connection graph” in Fig. 4. The “connection graph” only shows if a biomarker is affected and if this effect is positive or negative. Future studies will be required to quantify the effect of each biomarker individually on the risk for CHD. Furthermore when considering the implications of antidepressant use on the biomarkers of CHD it is important to note that antidepressants would likely only prove beneficial in patients with depression and not in the general population [65, 82].

It must be noted that like all pharmacotherapeutic therapies there is always the possibility for some adverse effects [83–85] and possible drug interactions [86]. However, SSRI treatment has proved to be both safe and effective in treating depression in patients with CHD [87].

## Discussion

The ‘connection graph’ for depression presented in Fig. 3 indicates that the effect of depression on CHD pathogenesis, as measured by effects on serological biomarkers of CHD, would likely serve to increase a depressed patients risk for CHD. The magnitude of this effect can be quantified through determining the RR for CHD offered by depression.

Observational studies considering the incidence of CHD in depressed patients may provide these answers. A meta-analysis of such studies comprising 124,509 patients in 21 studies found that the depressed had an increased RR for CHD of 1.90 (1.49 to 2.42) compared to healthy controls [4].

It is known that antidepressants such as SSRI’s can mediate the symptoms of depression [88] and impact the biomarkers of CHD in such a manner that would appear to be positive in terms of CHD risk (Fig. 4). Again the magnitude of this effect is evident in the potential reduction in CHD risk due to SSRI’s use in a depressed population initially without CHD [7].

In an observational study of 93,653 patients with depression, without CHD, it was found that patients, who had 12 or more weeks of antidepressant treatment, had a RR for CHD of 0.48 (0.44 to 0.52) compared to patients not treated. When using our risk presentation this equates to a possible 2.08-fold reduction in CHD risk. The observational nature of this study must be noted and conclusions on treatment cannot be directly drawn from these results. The results may allude to primary prevention of CHD due to SSRI use in the depressed [7].

Some of the important aspects of depression may be the increase in inflammation and dysregulation of metabolism evident through the increases in inflammatory and metabolic biomarkers [15, 47, 48, 89]. Comparing the ‘connection graphs’ of depression and SSRI use it is clear that some of the manners in which depression effects the serological biomarkers are mediated by SSRI’s.

These effects include positive impacts on coagulation, oxidative stress and metabolism which are deregulated by depression. The effects of depression on lipids are not wholly clear (Fig. 3) and accordingly the effects of SSRI’s on these would most likely not account for the decreased risk observed (Fig. 4).

Interestingly the inflammatory biomarkers which are all negatively influenced by depression are positively mediated by SSRI usage. This may highlight the importance of inflammation in the pathogenesis of CHD especially in how depression influences it. A combination of these changes presents the possible action of a risk reduction, such as those observed in depressed patients using SSRI’s [7].

The data from Fig. 3 and Fig. 4 show that inflammation and metabolic dysregulation may be key aspects in the pathogenesis of CHD [15, 45, 46, 90]. These aspects increase in depression and may play a part in the 1.90-fold increased risk for CHD. With the use of SSRI antidepressants these factors decrease and may present up to a 2.08-fold reduction in CHD risk. This further highlights the importance of inflammation and metabolic dysregulation the pathogenesis of CHD.

Depression not only has direct effects but can have further negative effects on the treatment and secondary prevention of CHD. Depressed patients typically have trouble adhering to medication and intervention therapy [91]. This could serve to explain some of the increased risk that is associated with depression after a CHD event [92]. These and direct actions of depression on CHD adds credence to the recommendation that depression should be elevated to the status of risk factor for poor prognosis in patients with CHD [93].

Based on the evidence we believe that the CHD risk associated with depression is substantial and should garner a similar level of public interest as does other risk factors such as smoking, high cholesterol and treatments such as statin therapy. We agree very strongly with recommendations presented by the American Heart Association that depression should be screened for regardless of a causal link between improved depression and CHD risk [94].

Further research is required in the form of adequately powered interventional trials on the efficacy of SSRI’s in primary prevention of CHD in depressed patients. Additionally, studies are required to determine the risk for CHD that would be associated with decreased serum levels of BDNF.

## Conclusions

It is apparent that depression has a wide ranging impact on the pathogenesis of CHD with these effects notable in changes in CHD biomarkers. However, depression can be mediated through the use of antidepressants such as SSRI’s. These antidepressants may mediate some of the negative pathogenetic effects of depression on CHD. Such effects are noted in the normalization of the CHD biomarkers in patients using SSRI’s. These effects result in a decreased risk for CHD observed in depressed patients using SSRI antidepressants.
